# Autodissemination of pyriproxyfen suppresses stable populations of *Anopheles arabiensis* under semi-controlled settings

**DOI:** 10.1186/s12936-019-2803-1

**Published:** 2019-05-09

**Authors:** Dickson Lwetoijera, Samson Kiware, Fredros Okumu, Gregor J. Devine, Silas Majambere

**Affiliations:** 1 Environmental Health and Ecological Sciences Department, Ifakara Health Institute, PO Box 53, Ifakara, Tanzania; 2 Department of Mathematics, Statistics and Computer Science, Marquette University, Milwaukee, WI USA; 3 Institutes of Biodiversity, Animal Health and Comparative Medicine, University of Glasgow, Glasgow, G12, 8QQ UK; 4 School of Public Health, Faculty of Health Sciences, University of the Witwatersrand, Johannesburg, South Africa; 5 QIMR Berghofer Medical Research Institute, Brisbane, QLD Australia; 6 Pan-African Mosquito Control Association, Nairobi, Kenya

**Keywords:** Autodissemination, Pyriproxyfen, Clay pots, Malaria vectors, *Anopheles arabiensis*, Semi-field, Ifakara, Tanzania

## Abstract

**Background:**

Autodissemination of pyriproxyfen (PPF), i.e. co-opting adult female mosquitoes to transfer the insect growth regulator, pyriproxyfen (PPF) to their aquatic habitats has been demonstrated for *Aedes* and *Anopheles* mosquitoes. This approach, could potentially enable high coverage of aquatic mosquito habitats, including those hard to locate or reach via conventional larviciding. This study demonstrated impacts of autodissemination in crashing a stable and self-sustaining population of the malaria vector, *Anopheles arabiensis* under semi-field conditions in Tanzania.

**Methods:**

Self-propagating populations of *An. arabiensis* were established inside large semi-field cages. Larvae fed on naturally occurring food in 20 aquatic habitats in two study chambers (9.6 × 9.6 m each), while emerging adults fed on tethered cattle. The mosquito population was monitored using emergence traps and human landing catches, each time returning captured adults into the chambers. Once the population was stable (after 23 filial generations), PPF dissemination devices (i.e. four clay pots each treated with 0.2–0.3 g PPF) were introduced into one of the chambers (treatment) and their impact monitored in parallel with untreated chamber (control).

**Results:**

Daily adult emergence was similar between control and treatment chambers, with average (± SE) of 14.22 ± 0.70 and 12.62 ± 0.74 mosquitoes/trap, respectively, before treatment. Three months post-treatment, mean number of adult *An. arabiensis* emerging from the habitats was 5.22 ± 0.42 in control and 0.14 ± 0.04 in treatment chambers. This was equivalent to > 97% suppression in treatment chamber without re-treatment of the clay pots. Similarly, the number of mosquitoes attempting to bite volunteers inside the treatment chamber decreased to zero, 6 months post-exposure (i.e. 100% suppression). In contrast, biting rates in control rose to 53.75 ± 3.07 per volunteer over the same period.

**Conclusion:**

These findings demonstrate effective suppression of stable populations of malaria vectors using a small number of simple autodissemination devices, from which adult mosquitoes propagated pyriproxyfen to contaminate aquatic habitats in the system. This is the first proof that autodissemination can amplify treatment coverage and deplete malaria vector populations. Field trials are necessary to validate these results, and assess impact of autodissemination as a complementary malaria intervention.

## Background

Long-lasting insecticide-treated nets (LLINs) and indoor residual spraying (IRS) remain the foremost strategies for malaria vector control, and the most efficient in controlling anthropophagic and endophilic vector populations in Africa [1, 2]. However, these interventions are imperfect in controlling mosquitoes that have natural or evolved behaviours that allow them to avoid lethal contact with LLINs and IRS [3]. In addition, it has been illustrated mathematically that due to challenges such as increased outdoor-biting and insecticide resistance, malaria elimination cannot be achieved even with extensive coverage of LLINs and IRS, mass screening and treatment combined [4, 5].

Treating mosquito aquatic habitats with larvicides can effectively reduce population densities of malaria vectors and associated malaria transmission [6]. This has the potential to complement LLINs and IRS especially in the settings where resistance to pyrethroids threatens LLINs and IRS [7, 8] and outdoor malaria transmission continues [9, 10]. Despite its potential effectiveness, larviciding, is only recommended for uses in settings where aquatic habitats are few, fixed and findable [11], which happen to be mostly urban. On the contrary, deployment of larviciding in rural settings requires strategies that efficiently target different habitats at high coverage and relatively less cost compared to LLINs and IRS interventions [12, 13].

The utility of autodissemination of PPF has been explored using adult female malaria vectors, under semi-field conditions [14, 15] and *Aedes* mosquitoes under field settings [16–18]. The technique uses mosquitoes that are seeking resting and oviposition sites to contaminate their aquatic habitats with PPF rendering those habitats unproductive. It offers a promising approach for wide scale deployment of larviciding programmes.

Though PPF is metabolized in mosquitoes by the same mechanism as pyrethroids [19], there is still no evidence of resistance development to PPF in malaria vectors [20] therefore this strategy may have potential to control pyrethroid susceptible and resistant malaria vectors when disseminated to their aquatic habitats [14, 21]. When deployed as a conventional larvicide, by vector control teams, the impact of PPF in the aquatic habitat can prevent adult emergence for up to 6 months under field settings, depending on formulation of PPF and nature of targeted habitats [21]. PPF also has the added advantage of low mammalian toxicity and short-lived impacts on non-target organisms [22, 23]. It is approved by the WHO for public health uses including mosquito control and it is safe in drinking water [24].

As the prerequisite for successful scaling up, autodissemination of PPF intervention requires rigorous conceptualization and testing at laboratory and semi-field scales to generate baseline information that can guide future field trials. Previous studies conducted under laboratory settings, showed that autodissemination of PPF by malaria vectors occurs when mosquitoes are exposed while resting after bloodmeal [15, 25, 26]. These findings were further corroborated in semi-field studies using *Anopheles arabiensis*, in which autodissemination of PPF to aquatic habitats occurred when mosquitoes were exposed via clay pots while resting after bloodmeal [14]. However, these initial studies utilized exceptionally large numbers of released mosquitoes and small artificial aquatic habitats. There was also no opportunity to examine amplification in coverage, and impacts of PPF was evaluated over very short time scales (equivalent to a single gonotrophic cycle and contamination event). The studies, therefore, missed the potential cumulative impacts of repeated resting, oviposition, contamination and dissemination events.

This latest study exploits the natural behaviors of the target vector, and tests a model that might be replicated in the field. Self-sustaining colonies of *An. arabiensis* were established under semi-field conditions with large volume (15–400 L) larval habitats. The main objective was to demonstrate potential for the autodissemination technique to crash a large population using clay pots treated with PPF.

## Methods

### Study site and facilities

The study was done inside the semi-field systems at Ifakara Health Institute’s Mosquito City facility, located in Kining’ina village (8.11417 S, 36.67484 E), in rural southern Tanzania from January 2014 to March 2016. Details of the design, preparation and use of semi-field system have been provided previously [14, 27]. Measurements of temperature and relative humidity (RH) were obtained from data loggers (Tinytag TV-1500, Gemini Data Logger, UK) installed in both semi-field system chambers, from Jan 2014 to May 2015.

### Establishment of the self-sustaining colonies

Two self-sustaining colonies of *An. arabiensis* were established in two separate semi field system chambers by introducing 200 larvae (2nd and 3rd instar) per aquatic habitat. The mosquitoes were obtained from a laboratory colony that had been established using mosquitoes from local villages in rural Tanzania. The rearing procedures for the *An. arabiensis* colony have been described previously [14, 26].

**Fig. 1:**
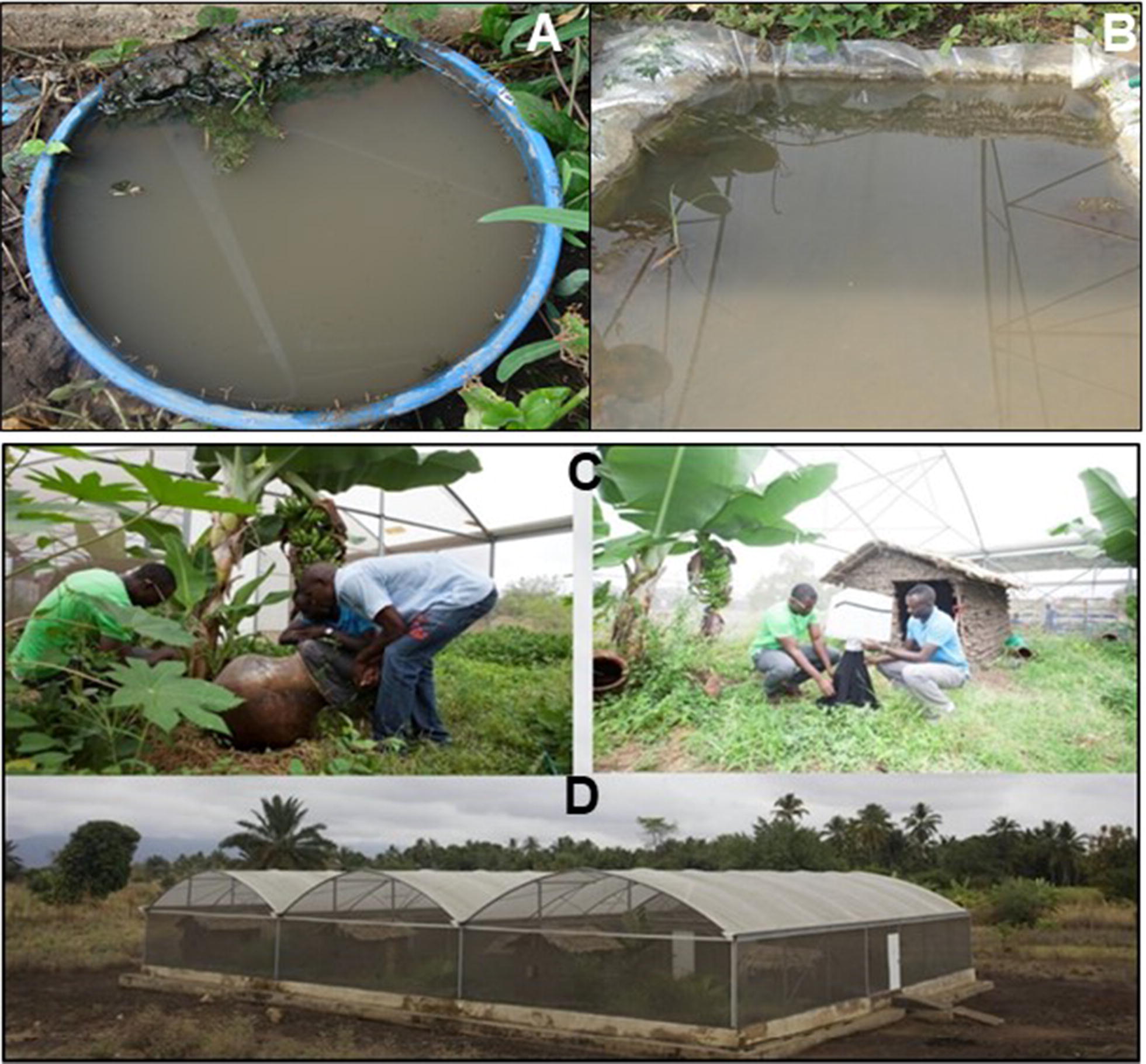
Pictorial representation of aquatic habitats, a plastic basin (**a**) and ground depression (**b**), installed inside semi-field system chambers (**d**), where mosquito self-sustaining colonies were established. The chambers had vegetation, a mud hut and mosquito resting sites, and cattle were let in every evening for mosquito to blood-feed on. Monitoring was done with emergence traps (**c**)

The aquatic habitats were made from twenty plastic basins (50 cm diameter and 15 L holding capacity each), half-filled with soil and other half with tap water and buried to the ground level around the edge of the basin inside the semi-field system (Fig. 1). The aquatic habitats were left for 2 weeks before introducing larvae into them, so as to allow settlement of soil segments and growth of algae necessary as larval food sources [28]. Water in the aquatic habitats was replenished to ensure its volume is kept at a relatively constant level. In addition, two large aquatic habitats with a surface area of 1.5 m^2^ (10 cm deep and 400 L holding capacity ground depression lined with impermeable plastic sheet and filled in with soil and tap water) were created in the middle of each semi-field system chamber to simulate larger, naturally occurring aquatic habitats. These large habitats were established 3 months after the self-sustaining populations were initiated so the larvae therein resulted from eggs deposited by the established population.

All larvae in the aquatic habitats fed on naturally forming algae and other nutrients present in the habitats, without adding any larval food supplement. To ensure that the emerged mosquitoes had bloodmeal sources, two cows were introduced and tethered inside a separate built mud huts in each semi-field system chamber daily at 16:00 h and moved outside at 07:00 h to graze freely.

### Participant safety and mosquito containment measures

All staff involved for the up-keeping of the self-sustaining colonies of *An. arabiensis* and performing human landing catches (HLC) were checked for malaria at weekly basis to ensure no malaria parasites were introduced into the self-sustaining colonies. Any staff detected with malaria was treated with recommended first-line medication (artemisinin-based combination therapy) for uncomplicated *P. falciparum* malaria [29]. Double doors were installed to prevent possible mosquito egress and ingress.

### Assessment of self-sustaining colonies before and after introducing pyriproxyfen

Before introducing PPF, baseline data on population growth and stability of the self-sustaining colonies collected through daily monitoring of the aquatic habitats for three consecutive months (March–May 2015) using emergence traps [30]. Six emergence traps were set on top of six randomly selected aquatic habitats daily. During the PPF-intervention (June 2015–March 2016) 12 of the 20 habitats were randomly selected and assigned into two groups of 6 habitats each for monitoring mosquito emergence. Six emergence traps, rotated between groups on a daily basis and were placed over one group of 6 habitats to measure the number of adult mosquitoes that emerged overnight. The remaining group of habitats was left open for mosquitoes to emerge and oviposit freely, but were fitted with the emergence traps the next day.

In the semi-field system’s treatment chamber, four clay pots lined with black cotton cloth that has been dampened with water and dusted with PPF powder were introduced. Each pot was treated with 2–3 g of a 10% AI PPF dust (Sumilarv^®^, Sumitomo Chemical Co. Ltd., Japan) by evenly spreading the PPF powder using painting blush over all surface of the dampen cloth inside the pot. Detailed procedures for treating pots have been described previously [14]. The intervention started with two clay pots for 2 months (June and July) and were increased to four pots. Four clay pots, all treated once, and at the same time, were used in the final trial design.

In both phases, before and after exposing mosquito colonies to PPF, all the trapped mosquitoes were counted and released back to the semi-field system. A cumulative sub-sample of 114 and 691 mosquitoes from collected before and after intervention respectively, were identified to species level using morphological keys and polymerase chain reaction [31, 32], to confirm the established colonies had not been contaminated by other species.

Human landing catches [33] were performed by two adult male volunteers sitting 8 m apart in each self-sustaining colonies chamber from 18:00 to 06:00 h on alternative days for 32 days in total. On days when no HLC took place, cows were introduced as a blood source for the population. Volunteers were rotated between positions and changed chambers every week.

Mosquito ovary dissection was performed to determine parity rate, which is the entomological indicator for mosquito longevity used to monitor and determine whether vector control intervention has been successful [34]. Additional cumulative sub-sample of 892 female *An. arabiensis* were collected between June and Dec 2015 killed by refrigeration and immediately dissected and categorized as nulliparous (mosquitoes that have neither blood-fed nor laid eggs) parous (those that have at least blood-fed once and laid eggs at least once).

Complete decimation of the mosquito population was confirmed by zero catches from emergence traps and HLC for 2 weeks (maximum developmental time from eggs to adults at normal tropical water temperatures) [35]. Observational assessment for the presence of any adult mosquitoes flying or resting around vegetation and inside mud huts where majority of mosquitoes prefer to rest was also conducted [14]. As a proxy indicator for continued oviposition events, presence but not abundance of larvae in the aquatic habitats was also monitored. In the fifth month post-treatment, the effective contamination of habitats by PPF was assessed by monitoring the residual effect of PPF in water samples from three randomly selected habitats, on second and third instar larvae from the insectary. For every habitat, three separate 200 mL of water samples in individual beakers were collected and, twenty larvae were introduced in each and monitored daily until all larvae and pupae had either died or developed and emerged to adults. Similar set up of PPF treatment was adopted for the control chamber, in which clay pots were not installed. In addition, measurements of temperature and relative humidity (RH) were obtained from data loggers (Tinytag TV-1500, Gemini Data Logger, UK) installed in both semi-field system chambers.

### Data analysis

Based on previously estimated average time of 22 days between consecutive generations of *An. arabiensis* under semi-field settings [30], the approximate number of generations was calculated as an indicator of mosquito population growth and stability. All data were analysed in R v2.12.2 [36] using *lme4* package [37] for generalized linear mixed effects models. The differences in the total number of *An. arabiensis* adults collected and proportion emerged between control and treatment groups were fitted to Poisson and binomial distribution respectively, and and relative rates (RR) with 95% confidence intervals calculated to estimate mean mosquito catches in a chamber with PPF, relative to the reference group (a chamber without PPF). Treatment groups (with/without PPF) were classified as fixed effect in the model, while total number of treated clay pots, number of aquatic habitats per control and treatment groups were assigned as random effects for the autodissemination of PPF and larval bioassay data. Similarly, the model was used to test whether proportions of *An. arabiensis* adults emerged between control and treatment chambers varied between sexes.

To estimate the proportion of surviving mosquitoes that had the greatest potential for malaria transmission, parous rates were calculated as number of parous females/number of females dissected [38]. Odds ratios were calculated to establish the association of the intervention on mosquito population age structure/parity rate. In addition, the average emergence inhibition (EI) from the larval bioassays was calculated from the number of laboratory larvae exposed and the overall emergence of adults. EI is calculated as: EI (%) = 100 − (T× 100)/(1/C). Where T = percentage of adult emerged from larvae exposed in water samples in the PPF-chamber, and C = percentage of adult emerged from control chamber [39].

## Results

From 2014 when the self-sustaining population was established, 23 generations by May 2015 before intervention was estimated, and 14 mosquito generations during PPF-intervention (June 2015–March 2016). During mosquito identification, all successful amplifications 90.4% (728/805) across experimental chambers were *An. arabiensis*. The remaining samples did not amplify (Table 1).

**Table 1:** Species composition of self-sustaining colonies amplified as *Anopheles arabiensis* across sampling period, before (^a^) and after intervention with pyriproxyfen

Sampling month, 2015	Proportion amplified as *An. arabiensis*		Total mosquitoes collected
	Control chamber	PPF chamber	
Mar^a^	0.78	1.00	18
Apr^a^	0.58	1.00	48
May^a^	0.91	1.00	48
Jun	0.96	0.86	48
Jul	0.90	0.96	159
Aug	0.93	0.88	161
Sep	0.95	0.91	161
Oct	0.81	0.96	162

Before PPF treatment, adult emergence between control and treatment chambers were similar with an average (± SE) of 14.22 ± 0.70 and 12.62 ± 0.74 mosquitoes respectively (*p*= 0.512). Following treatment, average adults emergence in a treatment chamber declined to 0.14 ± 0.04 within 3 months, and to zero mosquito across remaining 7 months of monitoring. Though not expected, the average adult population within a control chamber also declined to 5.22 ± 0.42 but rose to 5.73 ± 0.33 across same monitoring period. The reason for decline was attributed either to seasonal variation or the population stabilizing to its self-sustaining size.

**Fig. 2:**
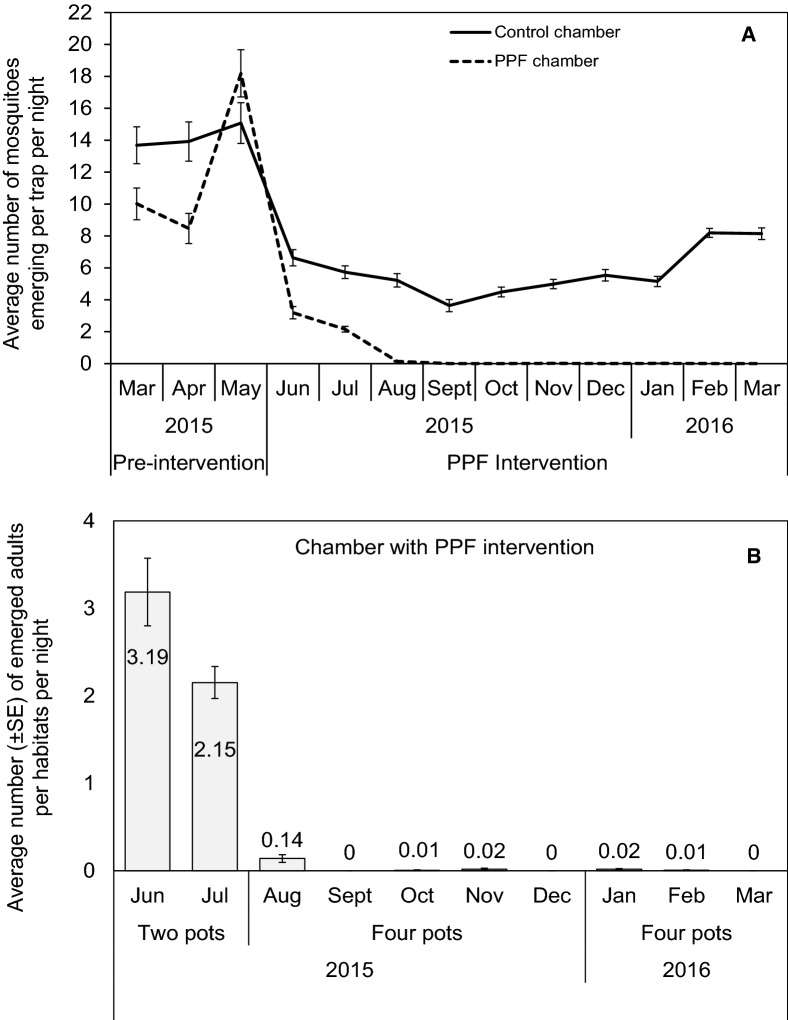
Average numbers of *Anopheles arabiensis* emerging over time before and during autodissemination intervention, from emergence traps (**a**), and with varying number of clay pots treated with pyriproxyfen (**b**)

In the PPF-chamber, *An. arabiensis* emergence success was significantly lower in female {Relative rates (RR) [95% confidence intervals] (CI) = 0.32 [0.31–0.33], *p *< 0.001)}, male (RR = 0.42 [0.40–0.44], *p *< 0.001), and both sex combined (RR = 0.36 [0.34–0.37], *p *< 0.001), compared to the control chamber, RR = 1 (Table 2). The decline of establish mosquito population depended upon the number of PPF-treated clay pots, whereby a mean number (±SE) of emerged adults per trap was 3.19 ± 0.39 and 2.15 ± 0.18 for month of June and July 2015 when two clay pots were installed, but less than one mosquito in the following months when four pots were installed (Fig. 2a, b).

**Table 2:** Estimated mean differences [and standard errors (SE)] in adult emergence of *Anopheles arabiensis* between control and pyriproxyfen exposed population during pyriproxyfen intervention

*Anopheles arabiensis*	Treatment	Total (N)	Mean ± SE	RR (95% CI)	p-values
Male	Control	5055	2.43 ± 0.15	1	
	PPF	2154	1.04 ± 0.14	0.42 (0.40–0.44)	< 0.001
Female	Control	9466	4.57 ± 0.22	1	
	PFF	3053	1.48 ± 0.20	0.32 (0.31–0.33)	< 0.001
Total	Control	14,521	7.00 ± 0.32	1	
	PFF	5207	2.52 ± 0.32	0.36 (0.34–0.37)	< 0.001

N = Total number of mosquitoes collected from emergence traps (n), 2073 in a control chamber and 2066 in a PPF chamber for the entire duration of the experiment, with estimated mean (N/n)

**Fig. 3:**
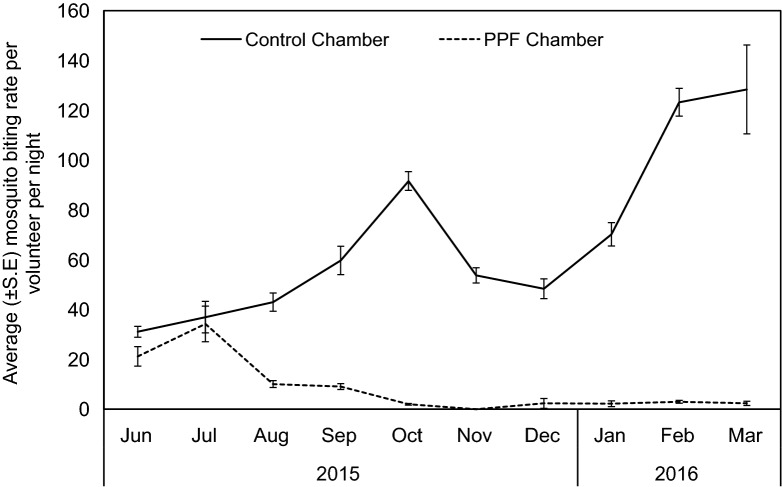
Average number of *An. arabiensis* mosquitoes collected by volunteers through human landing between pyriproxyfen-exposed and non-exposed population over time

During HLC, the mean number (± SE) of mosquitoes collected by volunteers from PPF-chamber per night was 8.21 ± 2.84, significantly lower (RR = 0.12 (0.11–0.13), *p *< 0.001) compared to the control chamber 68.65 ± 8.36 (Fig. 3). The percentage of parous mosquitoes in the PPF-chamber was 55% (224/406) compared to 26% (140/486) in the control chamber. The probability of catching a parous mosquito was approximately 4 times higher in a PPF-chamber (odds ratio (OR) [95% confidence intervals (CI)] = 3.88 [2.80–5.38], *p* ≪ 0.001) compared to control chamber, OR = 1.

The assessment of PPF residual effect in aquatic habitats on insectary reared larvae after 5 months post treatment demonstrated an emergence inhibition of 60.49%, with an average proportion (± SE) of 0.32 ± 0.08 adult emerged from a PPF chamber compared to 0.81 ± 0.06 from a control chamber (*p *< 0.0001).

The recorded daily average temperature and relative humidity within experimental chambers harbouring captive populations were similar. The average (± SD) minimum and maximum temperature recorded were 23.65 ± 5.34 °C and 30.75 ± 7.05 °C respectively, whereas average (± SD) relative humidity were 49.18 ± 38.47% and 84.80 ± 19.31% for minimum and maximum recordings respectively.

## Discussion

Building on documented evidence that malaria vectors *An. arabiensis* can successfully autodisseminate pyriproxyfen from treated surfaces [14], the current study, for the first time, demonstrates the unprecedented level of population control of the same vector species using the autodissemination strategy under semi-field settings. With > 97% reduction in adult emergence recorded within 3 months, complete population crash on the fourth month following mosquito exposure to pyriproxyfen was observed. In addition, HLC recorded approximately 94% and 100% reduction in host-seeking mosquitoes at fifth and seventh month of monitoring within the PPF-treated chamber probably due to combined effects of adult emergence inhibition at contaminated aquatic habitats [14], and sterilization of female mosquitoes with PPF [40].

Furthermore, the likelihood of collecting older (parous) mosquitoes was 4 times higher in PPF-exposed population compared to non-exposed. Contrary to adult based interventions that cause a shift to younger (nulliparous) mosquitoes due cumulative impacts on adult survival [41], the high proportion of parous mosquitoes in this study, indicates that the autodissemination technique, like other larviciding approaches, has a higher impact on younger mosquitoes (inhibition of emergence) and a limited impact on the survival of adults [42]. This imply that the surviving adult mosquitoes can promote successive transfer of PPF to aquatic habitats. This event is crucial for autodissemination as a “lure and release” strategy because it facilitate amplification of habitats coverage. Furthermore, this technique highlights the potential to even cover cryptic habitats that can neither be managed through conventional larviciding nor adulticiding campaigns.

Although no adult emergence was recorded on third month from water habitats, continuous presence of larvae in the aquatic habitats (Fig. 2a) and adult catches via HLC (Fig. 3) throughout the intervention monitoring phase highlighted continuing low level survival. One potential cause could be the reduction in residual activity of PPF at the aquatic habitats as the performed bioassay on water samples from the habitats using larvae from the insectary showed up to 32% adults emergence. While lower adult emergence confirm successful delivery of PPF to the habitats, low number of free-flying adults in the system could mean that not enough doses of PPF were transferred to habitats to prevent adults emergence. This control stage highlights a “self-limiting point” of the autodissemination technique under controlled condition [43], whereby remaining mosquito population are low and associated with marginal increase in emergence inhibition.

Hence, the maintained level of EI observed over 6 months period was likely due to multiple visit during the mosquito’s gonotrophic cycles that resulted into aquatic habitats coverage amplifications and accumulation of PPF at the habitats [44]. In addition, the presence of organic soil in habitats that adsorb PPF particles and cause its slow-release, might have also contributed in prolonged PPF persistence [22].

However, it is important to recognize the limitations of the current experimental design to inform future field-based trials. Firstly, although the impact of PPF on mosquito population was monitored over multiple mosquito generations at an extended period of time, the experiment was conducted once without replication owing to high cost and time of establishing stable colonies and semi-field system space limitations. Second, while clay pots used in this study are cheap, locally produced, and efficient in attracting blood fed mosquitoes [45], its fragility and unwieldiness present logistical challenges for a high coverage roll-out under field settings. Third, the size of the semi-field chambers meant that aquatic habitats and PPF treated clay pots were in close proximity and, therefore, easily accessible by PPF-carrying mosquitoes. In natural setting, wild malaria vectors might have to fly longer distances in search of oviposition sites when PPF particles carried by the mosquitoes might drop off or be groomed off, especially during dry season when habitats are few and farthest apart [44, 46].

Introducing autodissemination of PPF at field settings for controlling wild mosquito population, will require lighter, highly attractive, durable and easily to mass produce contamination devices. To achieve maximum transfer of PPF particles to high number of mosquitoes visiting the devices optimized PPF formulation will be desirable. For example, particles microencapsulation technology already in use to prolong residual efficacy of chemical insecticides and larvicides [47, 48], could also be used to optimize PPF formulation. On the other hand, the mosquito contamination devices with PPF can be augmented with desirable attractants for different mosquito physiological status to lure relative high numbers of mosquitoes into the device. Electrostatic materials that offer a high availability of insecticides to mosquitoes [49] might also be used to design an efficient and scalable autodissemination device. More importantly, it is envisaged that different mosquito species that coexist and share aquatic habitats, such as *Culex* and *Aedes* mosquitoes, might facilitate PPF delivery to the habitat and enhance amplification coverage of the aquatic habitats [44, 50]. With the increasing need to develop additional tools to sustain the gains already achieved by LLINs and IRS [2], autodissemination technique present a strong evidence for consideration as an outdoor based vector control strategy.

## Conclusion

These findings demonstrate effective suppression of stable populations of malaria vectors using a small number of simple autodissemination devices, from which adult mosquitoes propagated pyriproxyfen to contaminate aquatic habitats in the system. This is the first proof that autodissemination can amplify treatment coverage and deplete vector populations. Field trials are necessary to validate these results, and assess impact of autodissemination as a complementary malaria intervention.

## Data Availability

The original data from this study can be shared following the Ifakara Health Institute data sharing policy.

## Abbreviations

PPF: pyriproxyfen
LLINs: long-lasting insecticide-treated nets
IRS: indoor residual spray
EI: emergence inhibition
HLC: human landing catches
CI: confidence interval
OR: odds ratio
RR: relative rates

## References

[1] Bhatt S, Weiss D, Cameron E, Bisanzio D, Mappin B, Dalrymple U (2015). The effect of malaria control on *Plasmodium falciparum* in Africa between 2000 and 2015. Nature.

[2] WHO. World malaria report. Geneva: World Health Organization; 2018. http://www.who.int/iris/handle/10665/275867. Licence: CC BY-NC-SA 3.0 IGO.

[3] Kitau J, Oxborough RM, Tungu PK, Matowo J, Malima RC, Magesa SM (2012). Species shifts in the *Anopheles gambiae* complex: do LLINs successfully control *Anopheles arabiensis*?. PLoS One.

[4] Griffin J, Hollingsworth T, Okell L, Churcher T, White M, Hinsley W (2010). Reducing *Plasmodium falciparum* malaria transmission in Africa: a model-based evaluation of intervention strategies. PLoS Med..

[5] Rabinovich R, Drakeley C, Abdoulaye A, Djimde B, Fenton H, Simon I (2017). malERA: an updated research agenda for combination interventions and modelling in malaria elimination and eradication. PLoS Med..

[6] Tusting L, Thwing J, Sinclair D, Fillinger U, Gimnig J, Bonner KE (2013). Mosquito larval source management for controlling malaria. Cochrane Database Syst Rev..

[7] Hemingway J (2017). The way forward for vector control. Science.

[8] Strode C, Donegan S, Garner P, Enayati A, Hemingway J (2014). The impact of pyrethroid resistance on the efficacy of insecticide-treated bed nets against African Anopheline mosquitoes: systematic review and meta-analysis. PLoS Med..

[9] Russell T, Govella N, Azizi S, Drakeley C, Kachur SP, Killeen G (2011). Increased proportions of outdoor feeding among residual malaria vector populations following increased use of insecticide-treated nets in rural Tanzania. Malar J..

[10] Reddy MR, Overgaard HJ, Abaga S, Reddy VP, Caccone A, Kiszewski AE (2011). Outdoor host seeking behaviour of *Anopheles gambiae* mosquitoes following initiation of malaria vector control on Bioko Island, Equatorial Guinea. Malar J..

[11] WHO (2012). The role of larviciding for malaria control in sub-Saharan Africa.

[12] Worrall E, Fillinger U (2011). Large-scale use of mosquito larval source management for malaria control in Africa: a cost analysis. Malar J..

[13] Soper F, Wilson D (1943). *Anopheles gambiae* in Brazil: 1930 to 1940.

[14] Lwetoijera D, Harris C, Kiware S, Dongus S, Devine GJ, McCall PJ (2014). Effective autodissemination of pyriproxyfen to breeding sites by the exophilic malaria vector *Anopheles arabiensis* in semi-field settings in Tanzania. Malar J..

[15] Mbare O, Lindsay SW, Fillinger U (2014). Pyriproxyfen for mosquito control: female sterilization or horizontal transfer to oviposition substrates by *Anopheles gambiae* sensu stricto and *Culex quinquefasciatus*. Parasit Vectors..

[16] Devine GJ, Zamora Perea E, Killeen GF, Stancil JD, Clark SJ, Morrison AC (2009). Using adult mosquitoes to transfer insecticides to *Aedes aegypti* larval habitats. Proc Natl Acad Sci USA.

[17] Abad-Franch F, Zamora-Perea E, Ferraz G, Padilla-Torres SD, Luz SL (2015). Mosquito-disseminated pyriproxyfen yields high breeding-site coverage and boosts juvenile mosquito mortality at the neighborhood scale. PLoS Negl Trop Dis..

[18] Abad-Franch F, Zamora-Perea E, Luz SL (2017). Mosquito-disseminated insecticide for citywide vector control and its potential to block arbovirus epidemics: entomological observations and modeling results from Amazonian Brazil. PLoS Med..

[19] Yunta C, Grisales N, Nász S, Hemmings K, Pignatelli P, Voice M (2016). Pyriproxyfen is metabolized by P450s associated with pyrethroid resistance in *An. gambiae*. Insect Biochem Mol Biol..

[20] Invest JF, Lucas JR, Robinson WH, Bajomi DV (2008). Pyriproxyfen as a mosquito larvicide. Sixth international conference on urban pests.

[21] Kawada H, Dohara K, Shinjo G (1988). Laboratory and field evaluation of an insect growth regulator, 4-phenoxyphenyl (RS)-2-(2-pyridy1oxy)propyl ether, as a mosquito larvicide. Jpn J Sanit Zool..

[22] Schaefer CH, Miura EF, Dupras J, Mulligan F, Wilder WH (1988). Efficacy, nontarget effects, and chemical persistence of S-31 183, a promising mosquito (Diptera: Culicidae) control agent. J Econ Entomol..

[23] Sullivan JJ, Goh KS (2008). Environmental fate and properties of pyriproxyfen. J Pest Sci.

[24] WHO. Pyriproxyfen in drinking-water. Background document for preparation of WHO guidelines for drinking-water quality. Geneva: World Health Organization; 2008. WHO/HSE/AMR/08.03/10.

[25] Itoh T, Kawada H, Abe A, Eshita Y, Rongsriyam Y, Igarashi A (1994). Utilization of bloodfed females of *Aedes aegypti* as a vehicle for the transfer of the insect growth-regulator pyriproxyfen to larval habitats. J Am Mosq Control Assoc..

[26] Harris C, Lwetoijera D, Dongus S, Matowo NS, Lorenz LM, Devine GJ (2013). Sterilising effects of pyriproxyfen on *Anopheles arabiensis* and its potential use in malaria control. Parasit Vectors..

[27] Ferguson HM, Ng’habi KR, Walder T, Kadungula D, Moore SJ, Lyimo I (2008). Establishment of a large semi-field system for experimental study of African malaria vector ecology and control in Tanzania. Malar J..

[28] Gimnig JE, Ombok M, Otieno S, Kaufman MG, Vulule JM, Walker ED (2002). Density-dependent development of *Anopheles gambiae* (Diptera: Culicidae) larvae in artificial habitats. J Med Entomol.

[29] WHO (2015). Guidelines for the treatment of malaria.

[30] Ng’habi K, Mwasheshi D, Knols BGJ, Ferguson HM (2010). Establishment of a self-propagating population of the African malaria vector *Anopheles arabiensis* under semi-field conditions. Malar J..

[31] Gillies M, de Meillon B (1968). The Anophelini of Africa south of the Sahara (Ethiopian zoogeographical region).

[32] Scott JA, Brogdon WG, Collins FH (1993). Identification of single specimens of the *Anopheles gambiae* complex by the polymerase chain reaction. Am J Trop Med Hyg.

[33] Service MW (1977). Critical-review of procedures for sampling populations of adult mosquitos. Bull Entomol Res.

[34] Gillies MT (1954). The Recognition of age-groups within populations of *Anopheles gambiae* by the pre-gravid rate and the sporozoite rate. Annals Trop Med Parasitol..

[35] Gilles HM, Warrell DA, Service MW, Townson H (2002). The *Anopheles* vector. Essential Malariology.

[36] R Core Team. A language and environment for statistical computing. Vienna: R Foundation for Statistical Computing; 2013. http://www.R-project.org.

[37] Bates D, Maechler M, Bolker B. Linear mixed-effects models using S4 classes. 2013. Maintainer: lme4-author@R-forge.wu-wien.ac. http://lme4.r-forge.r-project.org/.

[38] WHO (2013). Malaria entomology and vector control.

[39] WHO (2018). Efficacy-testing of traps for control of *Aedes* spp. mosquito vectors.

[40] Lwetoijera DW, Harris C, Kiware SS, Killeen GF, Dongus S, Devine GJ (2014). Comprehensive sterilization of malaria vectors using pyriproxyfen: a step closer to malaria elimination. Am J Trop Med Hyg.

[41] Magesa S, Wilkes T, Mnzava A, Njunwa K, Myamba J, Kivuyo M (1991). Trial of pyrethroid impregnated bednets in an area of Tanzania holoendemic for malaria Part 2. Effects on the malaria vector population. Acta Trop..

[42] Charlwood JD, Tomás EV, Andegiorgish AK, Mihreteab S, LeClair C (2018). ‘We like it wet’: a comparison between dissection techniques for the assessment of parity in *Anopheles arabiensis* and determination of sac stage in mosquitoes alive or dead on collection. Peer J..

[43] Kiware SS, Corliss G, Merrill S, Lwetoijera DW, Devine G, Majambere S (2015). Predicting scenarios for successful autodissemination of pyriproxyfen by malaria vectors from their resting sites to aquatic habitats; description and simulation analysis of a field-parameterizable model. PLoS One.

[44] Devine GF, Killeen GF (2010). The potential of a new larviciding method for the control of malaria vectors. Malar J..

[45] Wong J, Bayoh N, Olang G, Killeen GF, Hamel MJ, Vulule JM (2013). Standardizing operational vector sampling techniques for measuring malaria transmission intensity: evaluation of six mosquito collection methods in western Kenya. Malar J..

[46] Charlwood JD, Vij R, Billingsley PF (2000). Dry season refugia of malaria-transmitting mosquitoes in a dry savannah zone of east Africa. Am J Trop Med Hyg.

[47] Mashauri FM, Manjurano A, Kinung’hi S, Martine J, Lyimo E, Kishamawe C (2017). Indoor residual spraying with micro-encapsulated pirimiphos-methyl (Actellic^®^ 300CS) against malaria vectors in the Lake Victoria basin, Tanzania. PLoS One..

[48] Michaelakis A, Papachristos DP, Rumbos CI, Athanassiou CG (2018). Effect of the combined application of microencapsulated synthetic oviposition pheromone (MSP) with different larvicidal agents on the oviposition of *Culex pipiens* biotype molestus. Pest Manag Sci.

[49] Andriessen R, Snetselaar J, Suer RA, Osinga AJ, Deschietere J, Lyimo IN (2015). Electrostatic coating enhances bioavailability of insecticides and breaks pyrethroid resistance in mosquitoes. Proc Natl Acad Sci USA.

[50] Ndenga BA, Simbauni JA, Mbugi JP, Githeko AK, Fillinger U (2011). Productivity of malaria vectors from different habitat types in the western Kenya highlands. PLoS ONE.

